# Issues and challenges in the assessment, diagnosis and treatment of cardiovascular risk factors: Assessing the needs of cardiologists

**DOI:** 10.1186/1472-6920-8-30

**Published:** 2008-05-16

**Authors:** Sean M Hayes, Martin Dupuis, Suzanne Murray

**Affiliations:** 1 AXDEV Group, 8 Place Commerce, Suite 210, Brossard, QC, J4W 3H2, Canada

## Abstract

**Background:**

This needs assessment, initiated by the Academy for Healthcare Education Inc. in cooperation with AXDEV Group Inc., explored the knowledge, attitudes, behavior, and skills of community-based and academic-affiliated U.S. cardiologists in the area of CV risk assessment, treatment, and management from July 2006 to December 2006.

**Methods:**

The needs assessment used a multistage, mixed-method approach to collect, analyze, and verify data from two independent sources. The exploratory phase collected data from a representative sampling of U.S. cardiologists by means of qualitative panel meetings, one-on-one interviews, and quantitative questionnaires. In the validation phase, 150 cardiologists from across the United States completed a quantitative online questionnaire. Data were analyzed with standardized statistical methods.

**Results:**

The needs assessment found that cardiologists have areas of weakness pertaining to their interpersonal skills, which may influence patient-physician communication and patient adherence. Cardiologists appeared to have little familiarity with or lend little credence to the concept of relative CV risk. In daily clinical practice, they faced challenges with regard to optimal patient outcome in areas of patient referral from primary-care providers, CV risk assessment and treatment, and patient monitoring. Community-based and academic-affiliated cardiologists appeared to be only moderately interested in educational interventions that pertain to CV risk-reduction strategies.

**Conclusion:**

Educational interventions that target cardiologists' interpersonal skills to enhance their efficacy may benefit community-based and academic-affiliated specialists. Other desirable educational initiatives should address gaps in the patient referral process, improve patient knowledge and understanding of their disease, and provide supportive educational tools and materials to enhance patient-physician communication.

## Background

The health and survival benefits of optimizing the treatment of cardiovascular disease (CVD) risk factors are undeniable, based on the results of both observational and randomized controlled trials [1]. Several meta-analyses and cost-benefit analyses of risk-factor reduction, including blood pressure (BP) reduction [2], cholesterol lowering [3,4], increased physical activity [5], glucose control in diabetics [6], weight loss among obese individuals [7], and smoking cessation [8,9] have revealed significant reductions in a wide variety of CVD endpoints, including myocardial infarction (MI) and stroke, health-care expenditures, and death [1].

The Framingham Heart Study and other studies have elucidated the quantitative relationship between CV risk factors and CHD [10]. These studies show that the major risk factors are additive in predictive power, but any major risk factor, if left untreated for many years, has the potential to produce CVD.

Knowledge about the detection, treatment, and control of cardiovascular (CV) risk factors provides the impetus and rationale for the prevention of cardiovascular disease (CVD)[1]. Effective prevention of CVD requires an adequate risk-factor assessment to categorize patients for the selection of appropriate therapeutic intervention [11]. In clinical practice, CV risk factors are identified and treated in those not yet ill (primary prevention) and among people with established CVD to prevent recurrent events (secondary prevention) by means of widely disseminated clinical practice guidelines [12,13]. Professional cardiology associations worldwide, including the American Heart Association (AHA) and American College of Cardiology (ACC), have established guidelines to stress the importance of CV risk-factor assessment, treatment, and management. However, virtually every study of preventive therapies has shown that physicians do not actively pursue the goals of prevention outlined in these guidelines [1].

With the emergence of outcome-based educational initiatives, needs assessments of practicing healthcare professionals have become a primary tool for identifying gaps and barriers to change in daily clinical practice. The rationale for a comprehensive needs assessment is to help those who provide educational and supportive programs to understand the what, how, and why of clinical decision-making behaviors. Analysis of a behavioral needs assessment reveals the differences between actual activities in daily clinical practice as compared to optimal clinical practices, i.e., "what is happening" versus "what should be happening", as indicated by evidence-based medicine, clinical practice guidelines, clinical research, and experts in the field, i.e., key opinion leaders.

The Academy for Healthcare Education Inc. embraced this research paradigm by partnering with Axdev Group Inc., an educational and performance research organization that actively supports healthcare research and educational initiatives, to conduct a comprehensive behavioral national needs assessment of practicing U.S. community-based and academic-affiliated cardiologists from July 2006 to December 2006.

The objective of this research initiative was to assess the clinical approaches of practicing cardiologists to CV risk-factor assessment, treatment, and management in order to identify knowledge gaps and major barriers to change that interfere with the adoption of preventive strategies in daily clinical practice. The goals was to provide specific, targeted recommendations to educational program developers to aid in the design of effective educational programs for practicing cardiologists to enhance best clinical practices in CV risk assessment, treatment, and management. A sub-research objective of this study was to examine the role of clinical practice guidelines in the assessment, treatment, and management of CV risk factors and to understand the extent to which cardiologists practice "beyond" or "outside" of guidelines.

## Methods

This needs assessment employed an applied cognitive behavioral design that embodies triangulation [14,15]. This design ensures the reliability and trustworthiness of the assessment by integrating qualitative and quantitative data-collection techniques in a mixed-methods approach (Table 1). Triangulation is defined as a method of research design that strengthens the study by using a combination of methodologies in the examination of the same phenomena. Overall, the rationale behind the mixed-methods approach is based on the logic that no single data-collection method adequately solves the problem of rival causal factors [16,17]. Because each method reveals different aspects of empirical reality, multiple methods of assessment are employed to ensure the trustworthiness of findings.

**Table 1:** Mixed methods approach

Study design	▪ A literature search and environmental scan were conducted to inform the design of the study and guide the development of the qualitative and quantitative research tools.
	▪ Research design and rationale, research tools, participant informed consent processes, and materials were submitted for ethics review to an Institutional Review Board (IRB). The ethics review was approved June 2006 (exploratory phase) and December 2006 (validation phase).
Exploratory phase (Qualitative)	▪ Panel meetings and key informant interviews were conducted with two selectively sampled groups of participants (actively practicing, academic-affiliated, and community-based cardiologists).

Validation phase (Quantitative)	▪ Online quantitative questionnaire, informed by the exploratory phase, was distributed to a national sample of actively practicing cardiologists.

Analysis	▪ Analysis, interpretation, conclusions, and recommendations based on multidisciplinary team collaboration and input.

The exploratory phase of data collection relied on semi-structured, qualitative, data-collection techniques and quantitative self-completion questionnaires among a small, representative sample of practicing community-based and academic-affiliated cardiologists. The validation phase employed a standardized online questionnaire, which was designed on the basis of findings from the exploratory phase. During the validation phase, strict sampling procedures were used to ensure a representative sample of practicing U.S. cardiologists.

### Sampling

Data collection during the exploratory phase occurred at full-day panel meetings in New York City, which were conducted with two homogenous groups of participants: academic-affiliated cardiologists (n = 11), who practice primarily in teaching hospitals, and community-based cardiologists (n = 9), who work primarily in non-teaching hospitals (Table 2). The majority of the participating cardiologists currently practice in the New England and Middle Atlantic regions.

**Table 2:** Study participants

	**Academic-affiliated cardiologists**	**Community-based cardiologists**	**Total**
**Exploratory phase**			
Full-day meetings	11	9	**20**
Key informant interviews	5	12	**17**
**Validation phase**			
Online survey	75	75	**150**

**Total**	**91**	**96**	**n = 187**

The panel meetings were semi-structured and included (a) democratic group discussion regarding cardiologists' roles; their issues and challenges in CV risk assessment, treatment, and management; CV residual risk; and continuing professional development (CPD) needs; (b) nominal group processes to establish cardiologists' priorities to enhance their clinical practice; and (c) a self-completion questionnaire designed to explore their learning needs, preferred learning formats, and perceived barriers to optimal CV risk assessment, treatment, and management. Throughout the process, the participants were asked to reflect upon their issues, challenges, and barriers in the continuum of care for patients with CV risk factors.

In addition to the full-day meetings, key informant (KI) interviews were conducted with an additional sampling of cardiologists (5 academic-affiliated cardiologists and 12 community-based cardiologists) from across the country. This sampling served to cross-validate the findings of the full-day meetings. The KI interviews followed a format similar to the panel meetings, but participants were interviewed in isolation. The semi-structured, audiotaped telephone KI interviews explored the same themes as the panel meetings. The participants of panel meetings and key informant interviews also completed a quantitative questionnaire.

The findings from the exploratory phase were used to guide the development of an online quantitative questionnaire, which was used during the validation phase. In this phase, 75 academic-affiliated cardiologists and 75 community-based cardiologists completed the online survey. These cardiologists were equally distributed across the country to ensure representation from the four major regions of the USA and were sampled to represent cardiologists who actively treat and manage patients with CV risk factors (Table 3). The participants responded to quantitative questions about gaps and barriers in CV risk assessment, treatment, and management that were identified in the exploratory phase and to assess their readiness to change.

**Table 3:** Regional distribution

	**New England and Mid-Atlantic**	**South Atlantic, East South Central, and West South Central**	**Pacific and Mountain**	**East North Central and West North Central**	**Total**
**Exploratory Phase**					

Panel meetings	19	1	-	-	**20**
KI interviews	4	6	4	3	**17**

**Validation Phase**					

Online survey	38	38	37	37	**150**

**Total**	**61**	**45**	**41**	**40**	**187**

### Data collection and analysis

Qualitative and quantitative data were collected by four distinct methods: panel meetings, individual interviews, self-completion forms, and an online survey. The resulting data were examined by multiple methods of analysis. Qualitative data from panel meetings were captured via flip charts and audiotapes, which were transposed by independent transcribers. All data were then categorized; axial and multidisciplinary coding was applied and tabulated by structured qualitative methods. The self-completion forms and data from the online survey were statistically analyzed by SPSS software, which assessed frequencies, t-tests, cross tabulations, and gap analyses.

## Results

The multidisciplinary analysis of findings from this needs assessment revealed that cardiologists face substantive challenges, issues, and gaps in CV-risk assessment, treatment, and management in their daily clinical practice, including treating across the continuum of care; the clinical application of professional guidelines; the management of CV residual risk; the patient-doctor relationship and patient adherence; and in their professional development needs.

### Professional challenges along the continuum of care

#### Referrals from primary-care physicians

According to cardiologists, substantive contributing factors in the referral process prevent the achievement of optimal patient health outcomes. They reported that patient referrals from primary-care physicians (PCPs) range from ambiguous to lacking in critical information; hence, the quality of PCP referrals was marred by distrust. They reported that PCP referrals are often inappropriately timed, e.g., too early or too late in the progression of CV risk factors, and lack clarity as to purpose or expected patient outcome.

**Figure 1:**
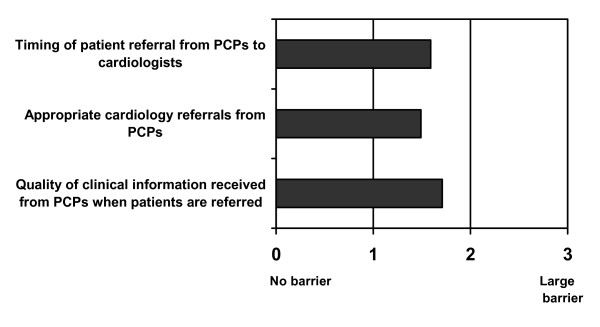
Barriers to positive health outcomes: referrals from primary-care physicians (n = 150).

The cardiologists reported that they receive two major types of inappropriate referral from PCPs: at-risk patients who could easily be cared for in primary practice; and complex patients, whom PCPs have little commitment or interest in managing due to the time investment that is required for patient management, although their training allows them to do so. The cardiologists also reported that PCPs delay appropriate referrals until patient health has substantively deteriorated; therefore, cardiologists receive these patients too late to impact their health outcome (Figure 1).

The quality of clinical information that cardiologists receive from PCPs was perceived as rarely optimal. In referral letters, patient history is often vague, the rationale for the referral is not always clearly communicated, and the patients themselves are often not informed and are therefore cannot explain the reason for their referral. Both academic-affiliated and community-based cardiologists shared these perceptions.

To summarize, cardiologists perceived the processes currently in place to facilitate their collaboration with PCPs as barriers to optimal clinical performance, as exemplified by the following statements.

*"They're [referrals] inappropriate because the physician [PCP] hasn't done what should have been done. Let me say this, if you look at it like fruit, this is an unripe referral."* Community-based cardiologist.

*"Frequently, you're just getting a piece of paper that says the diagnosis and you have no other idea of [why] the patient [is referred]."* Community-based cardiologist.

*"If you don't like or trust that physician, it makes communication difficult, and why would you communicate with somebody you don't trust?"* Academic-affiliated cardiologist.

*"I have times where I'll call the primary care physician to say, 'What exactly do you want from me?"'* Academic-affiliated cardiologist.

#### Gaps in screening, assessment, and diagnosis

As a consequence of inconsistent referrals and communication challenges, the cardiologists felt that they are required to initiate a comprehensive reassessment of CV risk among PCP-referred patients, because they do not trust the value of referral information. In effect, they felt forced to assume a PCP's role, because they perceive that PCPs have an inadequate understanding of what constitutes an adequate risk evaluation.

The cardiologists reported that PCPs are rarely up-to-date on recent medical literature and clinical guidelines. In addition, PCPs were not perceived as being aggressive enough in setting and reaching treatment goals. For example, when reviewing antihypertensive therapy, the cardiologists reported that they often question PCP-initiated treatment regimens and combination therapies. As a result, cardiologists felt obliged to confront treatment gaps among PCP-referred patients. The following statement effectively summarizes these observations.

*"There's an enormous gap between what physicians [PCPs] should know and do and what they actually do. It's a very sad state of affairs."* Community cardiologist.

When asked to self-evaluate their clinical competencies, cardiologists were confident in their knowledge and ability to assess CV risk factors. They rated their current risk-assessment competencies as close to what they considered to be ideal clinical practice. Table 4 delineates the summary of seven clinical-assessment practices, wherein participants rated their current and desired competency levels by using a 5-point Likert scale. Cardiologists did not self-assess any areas of CV risk assessment as a substantive gap, although some trends emerged. For example, with respect to competencies in the assessment of conditional risk factors, cardiologists rated their competencies moderately lower. Conditional risk factors were defined by elevated serum triglycerides, small LDL particles, elevated serum homocysteine, elevated serum lipoprotein, prothrombotic factors (e.g., fibrogen), and inflammatory markers (e.g., C-reactive protein).

**Table 4:** Competency gaps* in CV risk assessment (n = 150)

	**Current**	**Desired**	**Gap**	**Academic Gap**	**Community Gap**
Estimating incremental risk incurred by conditional risk factors	3.84	4.59	**0.75**	0.83	0.67
Estimating cardiovascular risk in primary prevention	4.14	4.75	**0.61**	0.73	0.48
Estimating incremental risk incurred by predisposing risk factors	4.07	4.67	**0.60**	0.68	0.52
Identifying conditional cardiovascular risk factors	4.07	4.65	**0.59**	0.67	0.51
Estimating cardiovascular risk in secondary prevention	4.29	4.75	0.46	0.56	0.36
Identifying predisposing cardiovascular risk factors	4.41	4.78	0.37	0.45	0.29
Identifying major independent cardiovascular risk factors	4.49	4.79	0.30	0.37	0.23

Self-assessment scale: 1 to 5. 1 = Low competence and 5 = High competence.

*Competency gaps are identified when the self-assessment of current competency is substantively lower than desired competency. If the gap between desired and current competencies is >1, then the gap is considered as substantive [18].

Although cardiologists are confident about their ability to assess CV risk factors, they face barriers from the systems in which they practice. For example, they struggle to incorporate the rules imposed by health maintenance organizations (HMOs) or insurance companies, who have introduced certain limitations for CV risk-factor assessment in clinical practice. As one academic-affiliated cardiologist commented,

"We have to justify doing procedures in order to learn more about the patient's risk."

In addition, some cardiologists felt obliged to order diagnostic tests and more in-depth assessments principally for legal protection, rather than because these tests were medically appropriate or necessary.

#### Treatment

Cardiologists reported three primary challenges in treating and managing patients with CV risk factors: managing patients with multiple concomitant complications and CV risk factors; balancing the benefits and risks of complex drug regimens and modifying inappropriate drug regimens of PCP-referred patients; and patient-provider relational issues, particularly patient adherence to lifestyle changes and medications.

Treating complex patients poses significant challenges for cardiologists. They accepted these challenges, because they saw it as their clinical responsibility, as specialists, to manage these patients to achieve a balance between multiple concomitant complications and CV risk. Elderly patients were identified as particularly complex to manage because of the potential repercussions of reducing diastolic BP too significantly. A community-based cardiologist summarized this concern as follows: "If you talk to a neurologist, we really don't know what the optimum blood pressure for those people should be. So lowering too low on those older patients might cause some problems with their CNS function."

Cardiologists observed that the cost of treatment and the impact of formularies and healthcare plans on patient finances represent substantive barriers to optimal patient health outcomes. The cardiologists reported that they are often confronted with patients who cannot or are unwilling to afford the medical regimen that represent best clinical practice.

Cardiologists reported that medical challenges arise when drug-related side effects impair patient safety, especially in patients with multiple comorbidities who require complex therapeutic regimens. A community-based cardiologist pointed out: "You're trying to balance achieving normality in whatever parameters you're treating, against their ability to achieve normality, whether it's limited by side effects or interactions with other diseases."

Drug-related side effects also create relational challenges for cardiologists, as side effects may substantively affect the patient's willingness to engage in the treatment. The cardiologists reported that they are often placed in the position of having to sell or convince patients of the value of treatment recommendations, a role for which they are poorly trained and are uncomfortable.

The independent discussions of community-based and academic-affiliated cardiologists indicated subtle differences between these groups with respect to their treatment challenges. Community-based cardiologists expressed more concerns about unclear treatment guidelines, particularly for patients with multiple CV risk factors. As one community-based cardiologist noted, "If all the studies are pretty clear, why is there a 5-point or 10-point difference [in BP targets] and [why do] different countries have different settings in terms of the success to goal?" Although academic-affiliated cardiologists acknowledged some struggles with contradicting studies, they indicated that optimal health outcomes are undermined by their community-based colleagues' inconsistent understanding of the importance of targeting end-organ damage for prevention.

#### Patient management

When managing patients with CV risk factors, cardiologists appeared to face knowledge gaps in the existing science base, contextual barriers, and legal concerns that interfere with optimal patient health outcomes.

They reported that a substantive barrier to optimal patient management is the lack of an effective means to monitor CVD progression. The majority described the current methods that are used to monitor CVD progression as suboptimal. They reported that the most practical means of evaluating the progression of end-organ damage is to assess kidney function with serum creatinine or urinalysis to detect albuminuria. They debated the clinical value of echocardiograms to evaluate left ventricular dysfunction and ultrasound evaluation of the carotid arteries, and they observed that cost limits the use of these technologies. Routine stress tests and the assessment of retinal vascularization were seen as optional means for monitoring the progression of CVD. A lack of skills and knowledge gaps in retinal evaluation were acknowledged as limitations to the use of this method.

The cardiologists indicated that long-term management of patients with CV risk factors is also complicated by reimbursement issues, which prevent them from monitoring patients as ideally as they would like, especially after the introduction of new therapies.

In terms of professional satisfaction, the cardiologists rarely acknowledged that they were not interested in monitoring "basic" hypertension, but they reported that many of their colleagues avoid this responsibility. Interestingly, academic-affiliated cardiologists pointed out that community-based cardiologists have little interest in managing hypertension, unless patients have other CV problems, while community-based cardiologists expressed similar views about their academic-affiliated peers.

### The role of clinical practice guidelines

Clinical practice guidelines were unanimously perceived as being important, and reaching the recommended treatment goals was seen as critical to improve patient health outcomes. Although cardiologists recognized that guidelines are used as benchmarks for appropriate standards of care, there were widely differing opinions about the genuine impact of guidelines on treatment targets.

During the exploratory phase, the cardiologists often commented that guidelines can oversimplify the clinical realities with which they are confronted to on a daily basis and do not integrate the most recent literature. As a result, while embracing the value of guidelines at a high level, most cardiologists were critical of the actual clinical value of guidelines in their daily practices. This finding was confirmed in the validation phase. Overall, the findings indicated that, although guidelines have imperfections and can become a source of frustration in daily practice, cardiologists believed in their clinical value for patient health outcomes.

Cardiologists reported that they do not treat as aggressively as recommended in guidelines when patients develop side effects that impact their quality of life or seem detrimental to their overall condition, e.g., when hypotensive episodes cause cerebral side effects, erectile dysfunction, or a rise in creatinine levels.

Following clinical guidelines serves as a source of legal protection, they indicated, as this practice provides documented evidence that they have aligned their clinical practices with the standard of care. They can use this evidence to overcome payers' minimum reimbursement criteria.

A sub-research objective of this study was to examine the role of clinical practice guidelines in the assessment, treatment, and management of CV risk factors and to understand the extent to which cardiologists practice "beyond" or "outside" of guidelines. These concepts were not predefined for participants. Although cardiologists' interpretation of these terms were not always exactly the same, most viewed "beyond" guidelines as meaning a more aggressive approach than recommended by guidelines, while "outside" guidelines meant making clinical decisions to manage the patient not in accordance with guidelines.

#### Beyond guidelines

Cardiologists reported that they routinely practice "beyond" guidelines and are more aggressive in their approach to treatment when recent clinical data, published after the distribution of guidelines, demonstrate the benefits of more aggressive targets than those delineated in the guidelines. They also reported that, because they often treat patients who are at higher risk of CVD than the typical patients in large clinical trials that support the development of guidelines, they aim for more aggressive targets in these patients.

#### Outside guidelines

Cardiologists acknowledged that they treat "outside" guidelines when treatment decisions must be more individualized. Specifically, they reported that individualized treatment goals must be set for patients whose ethnicity was not studied in clinical trials used to set treatment goals, for patients who are biologically resistant to medications, for elderly patients for whom less aggressive treatment goals must be set, and for patients who suffer from severe comorbidities, such as cancer, that are more likely to compromise a person's life expectancies than CV risk factors.

### Gaps in health outcomes due to risk assessment

Risk-reducing strategies can never completely eliminate nor control all CV risks. After any intervention, some level of risk remains. The concept of residual risk is used to describe this reality.

**Figure 2:**
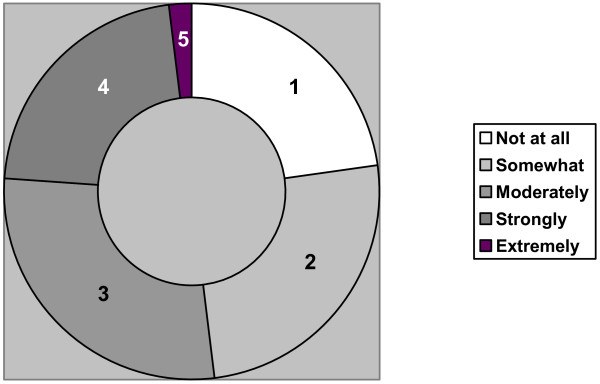
**Cardiologists' familiarity with the concept of "residual risk" in the context of CV risk management (n = 150)**. 1 = Not at all, 2 = Somewhat, 3 = Moderately, 4 = Strongly, 5 = Extremely. 76% of participants are "Not at all" to "Moderately" familiar with the notion of residual risk.

**Figure 3:**
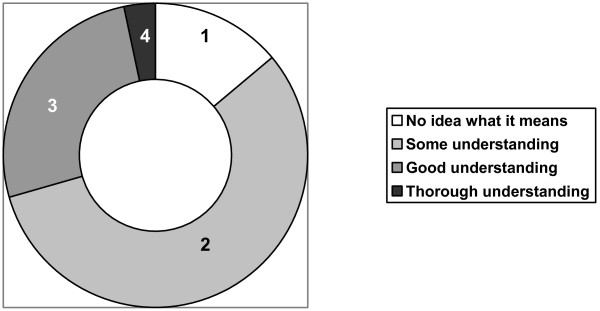
**Cardiologists' current understanding of the concept of "residual risk" in the context of CV risk management (n = 150)**. 1 = No idea what it means, 2 = Some understanding, 3 = Good understanding, 4 = Thorough understanding. 70% of participants have "No idea" to "Some understanding" of the concept of "residual risk".

The results of the exploratory phase showed that cardiologists are unfamiliar with and lack clear knowledge of what constitutes residual risk. This finding was subsequently validated in the online survey (Figures 2 and 3). The cardiologists reported that they rarely discuss residual risk in academic or clinical contexts. They proposed three definitions for this concept: the level of CV risk that remains despite BP stabilization; patients with controlled hypertension carry a higher risk than patients without hypertension; and people who have hypertension, even if controlled, have something biologically "wrong" with their CV system and are therefore at higher risk.

The cardiologists questioned the clinical value of residual risk, based on the lack of supportive data, e.g., evidence showing that a BP of 110/70 mm Hg is better than a BP of 120/80 mm Hg. They voiced concerns about the impact of more aggressive treatment on patients' quality of life. They were significantly concerned about the impact of treating residual risk on the patient-physician relationship and wondered whether they should discuss the implications of residual risk with their patients, as they felt that such discussions might discourage patient compliance.

### Gaps in health outcomes due to patient adherence

**Figure 4:**
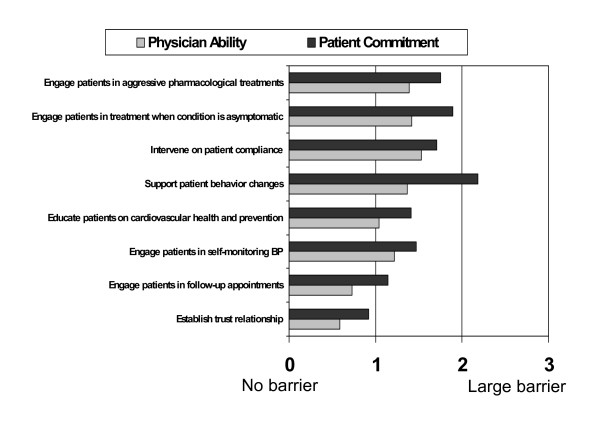
Barriers to positive health outcomes in the treatment and management of patients at risk for cardiovascular diseases: Cardiologists' ability and patients' commitment to enhance adherence (n = 150).

Patient adherence to treatment recommendations was viewed as a significant, often overriding challenge for cardiologists. They clearly perceived that patients are the primary source of challenge to achieving optimal patient health outcomes. They identified gaps in patient knowledge or understanding of their condition, patient commitment to risk-reducing behaviors and prescribed therapy, and their own ability to engage patients in risk-reducing strategies as barriers to optimal patient care (Figures 4). These barriers were not perceived as being insurmountable; however, the cardiologists felt that they had a significant impact on the patient-physician relationship. In addition, they observed that such relationships are becoming increasingly difficult, as patients are more informed and more involved in treatment decisions. They reported that a lack of transparency and accurate communication between themselves and patients are barriers to optimal care.

The cardiologists appeared to struggle with their own contribution to the patient-physician relationship and their role in patient adherence. When asked to assess to what extent they contributed to the physician-patient relationship and influenced patients by engaging them in treatment decisions, behavior change, or interventions to enhance adherence, they reported that patient commitment was a greater barrier to the attainment of these goals. They did not seem to recognize that their skills or abilities as healthcare professionals had an impact on the dynamics of patient adherence; instead, they clearly felt as if they were disempowered to alter these critical issues substantively and are thus subject to a patients' whims.

### Learning needs and commitment to professional development

Cardiologists expressed few if any perceived needs for education in the areas of assessment, diagnosis, treatment, and monitoring of patients with CV risk factors. Although only moderately satisfied with their practices in these areas of care, they did not believe that they needed to improve and were only moderately interested in actively pursuing educational activities.

## Discussion

The needs assessment found that cardiologists face several challenges along the continuum of care of patients with CV risk factors, particularly in the referral process. Similar findings were previously found where both primary care physicians and specialists perceived their mutual communication as inadequate and often absent [19,20]. The current study found additional challenges in risk assessment, treatment of patients with multiple risk factors and comorbidities, and patient monitoring. Cardiologists appeared to struggle substantively with their efforts to enhance patient adherence to CV risk-factor modification by medical therapy or lifestyle change.

Cardiologists demonstrate areas of weakness in core competencies as reflected by the American Board of Medical Specialists that involve patient care and interpersonal communication skills. Difficulty of physicians to discuss diagnosis, risks or bad news is well documented across all specialties [21,22]. Despite no evidence-based guidelines for communicating with patients, ways to support physicians communicating risk and evidence to patients have been identified [22]. However, a key finding of this needs assessment was that cardiologists often failed to acknowledge their own knowledge, skills, attitudes, and behaviors as barriers to optimal patient health outcomes. This finding is supported by the systematic review of literature conducted by Davis and colleagues in 2006 [23]. Their review of literature suggested that physicians have a limited ability to accurately self-assess their needs, knowledge, confidence, skills and competencies. This finding was independent of level of training, specialty, and domain assessed [23]. In the current study, cardiologists tended to believe that barriers to optimal health outcomes were solely derived from patients and PCPs, whose competencies in the area of CV management were often questioned. They appeared to underestimate the relevance of these factors to their own professional competency.

The finding that cardiologists are only moderately interested in educational activities poses a challenge for those who organize CPD events that aim to improve patient health outcomes. Historically, CPD providers have tended to develop educational interventions to improve medical knowledge as opposed to interpersonal or counseling skills, attitudes, and beliefs. Addressing attitudinal and skill-based needs demands innovative educational strategies.

## Conclusion

The findings of this needs assessment reveal numerous opportunities to generate a positive impact on patient health outcomes in the area of CV risk assessment and management. These opportunities include specific educational interventions, such as educational programs, materials and tools that enhance interprofessional and patient-cardiologist communications and that facilitate a better understanding of CVD among patients.

These findings indicate a need for targeted research into the perspectives of patients and PCPs on these important issues. Given the importance of the patient-physician relationship and its impact on long-term adherence, patient-based research about cardiologists' interpersonal and communication skills would provide critical data for designing educational initiatives that enhance the patient-cardiologist collaboration. CPD that incorporates strategies and techniques to build cardiologists' skills and confidence in addressing patient compliance, promote/increase concordance between cardiologists and patients, and addresses the cardiologist's responsibility for promoting better patient-physician collaboration would be valuable for practicing cardiologists. Specific educational interventions should:

• Clarify the value of engaging patients in a dialogue on adherence to their recommended therapeutic regimen.

• Be aligned with basic tenets of effective counseling and patient management, as outlined in the core competencies established by the American Board of Medical Specialists.

• Provide patient educational tools to address readiness to change/resistance among patients.

• Educate cardiologists about how patients think and about the scope of a physician's influence in engaging patients to adhere to treatment.

• Be directed toward facilitating patient-cardiologist interactions, interprovider communications, continuity of interaction, and sharing with family members.

• Shift the physician's focus of control through sessions that target beliefs and attitudes about patient vs. physician responsibility to increase physician confidence and impact patient collaboration.

In order to link educational interventions with patient health outcomes and professional satisfaction, these initiatives need to be pragmatic, easy to apply, and tied to measures of success for cardiologists and patients.

To strengthen interprofessional collaboration between cardiologists and PCPS, educational interventions should be targeted to build local or regional standards of practice that enhance communication, e.g., standardized referral tools, checklists, templates, etc. Research into the PCP perspective on patient referral would provide a more complete picture of current issues and challenges that impact patient health outcomes.

Patient education and understanding of CV risk and the importance of controlling major CV risk factors are critical to long-term adherence to medical therapy or lifestyle changes. Patient educational materials could take the form of storyboards on CV health, CV risk, or life-outcome scenarios. Such tools would help patients to feel in charge of their health and assume responsibility for adherence to therapy. These materials would facilitate cardiologists' influence on patient-health outcomes.

## Competing interests

The authors declare that they have no competing interests.

## Authors' contributions

SMH conceived and supervised the study in addition to participate in the data collection, data analysis, interpretation and review of manuscript. MD participated in the design of the study, data analysis, interpretation, review of manuscript and coordination of submission. SM participated in the design of the study, data collection and review of manuscript. All authors read and approved the final manuscript.

## Pre-publication history

The pre-publication history for this paper can be accessed here:


